# Predicting Proteolysis in Complex Proteomes Using Deep Learning

**DOI:** 10.3390/ijms22063071

**Published:** 2021-03-17

**Authors:** Matiss Ozols, Alexander Eckersley, Christopher I. Platt, Callum Stewart-McGuinness, Sarah A. Hibbert, Jerico Revote, Fuyi Li, Christopher E. M. Griffiths, Rachel E. B. Watson, Jiangning Song, Mike Bell, Michael J. Sherratt

**Affiliations:** 1 Division of Cell Matrix Biology & Regenerative Medicine, Faculty of Biology, Medicine and Health, Manchester Academic Health Science Centre, Manchester M13 9PT, UK; alexander.eckersley@manchester.ac.uk (A.E.); christopher.platt@manchester.ac.uk (C.I.P.); callum.stewart-mcguinness@postgrad.manchester.ac.uk (C.S.-M.); dr.sarah.hibbert@gmail.com (S.A.H.); 2 Monash Bioinformatics Platform, Monash University, Melbourne, VIC 3800, Australia; jerico.revote@monash.edu; 3 Infection and Immunity Program, Biomedicine Discovery Institute and Department of Biochemistry and Molecular Biology, Monash University, Melbourne, VIC 3800, Australia; jiangning.song@monash.edu; 4 Department of Microbiology and Immunology, The Peter Doherty Institute for Infection and Immunity, The University of Melbourne, Melbourne, VIC 3800, Australia; fuyi.li1@monash.edu; 5 Centre for Dermatology Research, Faculty of Biology, Medicine and Health, and Salford Royal NHS Foundation Trust, Manchester Academic Health Science Centre, Manchester M13 9PT, UK; Christopher.Griffiths@manchester.ac.uk (C.E.M.G.); Rachel.Watson@manchester.ac.uk (R.E.B.W.); 6 NIHR Manchester Biomedical Research Centre, Central Manchester University Hospitals NHS Foundation Trust, Manchester Academic Health Science Centre, Manchester M13 9WL, UK; 7 Monash Centre for Data Science, Faculty of Information Technology, Monash University, Melbourne, VIC 3800, Australia; 8 Research and Development, Walgreens Boots Alliance, Thane Road, Nottingham NG90 1BS, UK; Mike.Bell@boots.co.uk

**Keywords:** extracellular matrix, protease, aging, skin, deep-learning, machine learning, biomarkers, degradomics

## Abstract

Both protease- and reactive oxygen species (ROS)-mediated proteolysis are thought to be key effectors of tissue remodeling. We have previously shown that comparison of amino acid composition can predict the differential susceptibilities of proteins to photo-oxidation. However, predicting protein susceptibility to endogenous proteases remains challenging. Here, we aim to develop bioinformatics tools to (i) predict cleavage site locations (and hence putative protein susceptibilities) and (ii) compare the predicted vulnerabilities of skin proteins to protease- and ROS-mediated proteolysis. The first goal of this study was to experimentally evaluate the ability of existing protease cleavage site prediction models (PROSPER and DeepCleave) to identify experimentally determined MMP9 cleavage sites in two purified proteins and in a complex human dermal fibroblast-derived extracellular matrix (ECM) proteome. We subsequently developed deep bidirectional recurrent neural network (BRNN) models to predict cleavage sites for 14 tissue proteases. The predictions of the new models were tested against experimental datasets and combined with amino acid composition analysis (to predict ultraviolet radiation (UVR)/ROS susceptibility) in a new web app: the Manchester proteome susceptibility calculator (MPSC). The BRNN models performed better in predicting cleavage sites in native dermal ECM proteins than existing models (DeepCleave and PROSPER), and application of MPSC to the skin proteome suggests that: compared with the elastic fiber network, fibrillar collagens may be susceptible primarily to protease-mediated proteolysis. We also identify additional putative targets of oxidative damage (dermatopontin, fibulins and defensins) and protease action (laminins and nidogen). MPSC has the potential to identify potential targets of proteolysis in disparate tissues and disease states.

## 1. Introduction

Although the causative mechanisms of aging are not yet fully understood, there is compelling evidence that biochemical pathways, including protein oxidation, and protease-mediated cleavage, contribute to proteolysis and loss of proteostasis [1]. Similarly, oxidative stress and aberrant protease activity are also implicated in pathological remodeling of both acute and chronically inflamed tissues [2,3,4]. In the skin, for example, ultraviolet radiation (UVR), oxidative stress, and upregulated protease activity are interlinked processes associated with clinical photoaging, which manifests in the dermis as profound histological remodeling of fibrillar collagens and elastic fibers and the accumulation of oxidative damage [5,6,7,8]. Although collectively UVR, ROS and protease-driven proteolysis may contribute to the production of the skin photoaging degradome, the relative importance and specific targets of action of these different mechanisms remain poorly defined [9]. A better understanding of tissue proteolysis in the dermis and other tissues may lead to the identification of novel candidate biomarkers of damage and therapeutic targets [10,11].

Previous studies from our group suggest that both the in vivo remodeling of elastic fiber-associated fibrillin microfibrils, which is a hallmark of early photoaging, and the relative susceptibility of these assemblies to in vitro UVR, is likely to be due to specific amino acid (AA) compositions of the component proteins, principally fibrillin-1 [7,12,13]. This protein, and others also enriched in both UVR-absorbing (UV-chromophore) and oxidation-sensitive amino acid (AA) residues, are susceptible to degradation by environmentally attainable UVR doses [14]. In contrast, ECM protein assemblies, such as collagens I and VI and tropoelastin, are relatively resistant (as determined by electrophoretic mobility or ultrastructure) to these doses and wavelengths [13]. Recently, we have also demonstrated, using a newly-developed proteomic peptide location fingerprinting methodology, that UVR exposure can induce subtle structure-associated changes in the largest collagen VI alpha chain (alpha-3), which are not detectable as changes in global collagen VI microfibril ultrastructure [15] or in vivo architecture [16]. Therefore, AA composition appears to be a good predictor of relative susceptibility to UVR and oxidative damage (which is also a factor in non-UVR exposed tissues) [17,18,19,20]. However, predicting the relative susceptibility of proteins to protease-mediated cleavage is a more difficult task, as enzymatic proteolysis is dependent not only on the primary structure (AA sequence), but also on higher-order structures [21]. Despite this complexity, there remains the possibility that (as with relative UVR chromophore abundance) protease cleavage site abundance may serve as a key determinant of relative protease susceptibility.

The matrix metalloproteinases (MMPs) are a large family of zinc-dependent enzymes, which are thought to play key roles in skin photoaging and many other age- and disease-related disorders [22]. Other enzyme families, such as serine proteases, can also degrade ECM proteins [23,24] but their action in skin aging is not as well characterized. In contrast, the validated prediction of cleavage sites of enzymes, such as trypsin (where cleavage occurs at the C-terminal side of Arg and Lys AA residues when not followed by Pro) and the *Staphylococcus aureus* protease V8-GluC (where cleavage occurs on the C-terminal side of Glu in preference to Asp) is well established [25,26]. However, the prediction of cleavage sites in ECM proteins by MMPs and cathepsins requires the development and application of complex mathematical models based on state-of-the-art machine learning and deep learning techniques, which utilize data available in databases, such as MEROPS [27,28]. A number of bioinformatic tools have been developed to predict cleavage sites in AA sequences. For example, PROSPER, which is based on support vector machines (SVMs) and DeepCleave, uses a deep convolutional neural network (CNN). Both utilize some protein structure features to perform their predictions [29,30,31,32]. However, to our knowledge, the predictions of these algorithms have not been experimentally validated against native ECM proteins, nor has their comparative performance been evaluated in this context. Moreover, although recurrent neural networks have shown promising results in protein sequence and function prediction [33], this architecture, again to our knowledge, has not previously been employed for proteolytic cleavage site prediction [34,35].

The first goal of this study was to experimentally determine the ability of PROSPER and DeepCleave to identify MMP9-determined cleavage sites in two exemplar purified proteins (decorin (DCN) and vitronectin (VTN)) and subsequently in a complex ECM proteome derived from cultured human dermal fibroblasts (HDFs). Given the relatively poor performance of these algorithms against native ECM proteins, our next aim was to develop and evaluate a new protease cleavage prediction algorithm: Manchester proteome cleave (MPC), which uses state-of-the-art deep bidirectional recurrent neural network (deep BRNN) architecture [34,35]. Finally, we integrated the MPC model with amino acid composition analysis to produce a new web tool, the Manchester proteome susceptibility calculator (MPSC), which can predict relative protein susceptibility based on AA and protease cleavage site abundance) to two major mechanisms of tissue proteolysis.

## 2. Results and Discussion

### 2.1. Accurate Prediction of Protease Cleavage Sites in Native ECM Proteins Is Challenging

**Figure 1:**
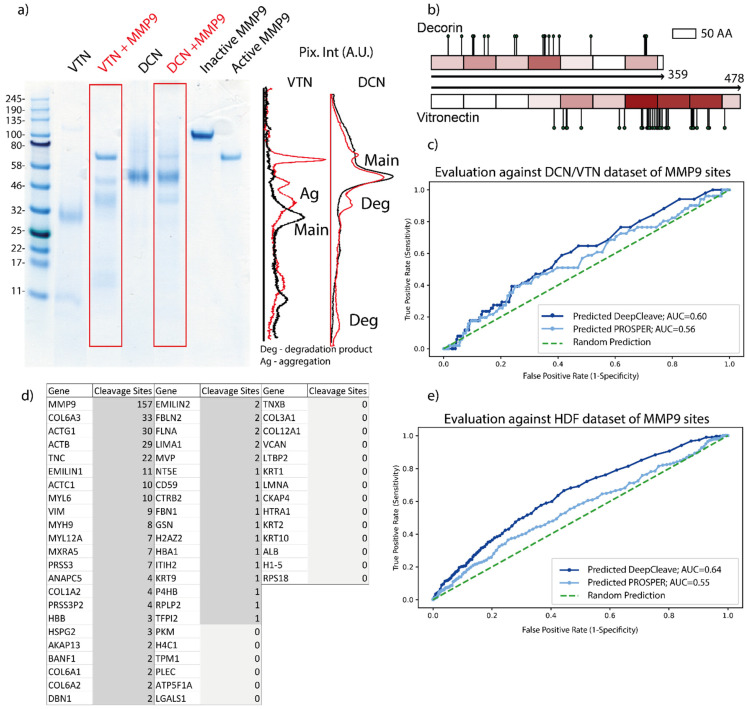
Prediction and validation of protease cleavage sites by existing models. (**a**) Gel-electrophoresis and associated densitometry profiles for control (black) and MMP9-digested (red) vitronectin (VTN) and decorin (DCN) proteins (the apparent Mw of glycosylated DCN is 50 kDa). MMP9 digestion induced profound changes in the electrophoretic profile of VTN. The single ~30 kDa monomer band was replaced by two higher Mw species. In contrast, MMP9 exposure decreased the staining intensity of the 50 kDa DCN monomer band and promoted the formation of putative lower Mw degradation products. (**b**) Differential degradation of VTN and DCN was confirmed by LC–MS/MS on bands excised from the same gel. Pins on the schematics represent experimentally determined cleavage sites, and the heat maps illustrate the number of experimental cleavage sites per 50 AA window (darker red = more sites per window). (**c**) receiver operating characteristic (ROC) curves evaluating experimentally detected MMP9 cleavage sites (by LC–MS/MS) from purified VTN and DCN proteins against protease cleavage site prediction models (PROSPER)- and DeepCleave-predicted cleavage sites. Both algorithms struggled to accurately predict experimental sites in native proteins. (**d**) MMP9-digested proteins detected by LC–MS/MS in a complex cell-derived matrix. A total of 60 proteins were detected, from which 40 had at least one non-tryptic cleavage site (assumed to be due to MMP9). (**e**) ROC curves evaluating experimentally detected MMP9 cleavage sites (by LC–MS/MS) from a complex cell-derived matrix against PROSPER- and DeepCleave-predicted cleavage.

In the skin, MMPs and other proteases, such as elastase and members of the cathepsin and granzyme families, play a significant role in tissue maintenance and remodeling [36,37]. Prediction of proteolytic cleavage sites is dependent on not only the 1° AA sequence but also the higher-order 2°–4° structures, which determine features, such as solvent accessibility and disordered regions [38,39]. Although currently available algorithms, such as PROSPER and DeepCleave, can predict cleavage sites with a good degree of accuracy [29,30], these predictions are (i) often limited to a specific subset of proteases and; (ii) lack experimental validation both in simple and complex model systems. In this study, we used SDS–PAGE and liquid chromatography–tandem mass spectrometry (LC–MS/MS) to characterize protease-mediated proteolysis in order to validate and compare protease cleavage site predictions for MMP9 (by PROSPER and DeepCleave) for purified DCN and VTN. MMP9 was chosen due to its association with the skin photoaging process [40]. By SDS-PAGE, we confirmed that both DCN and VTN are MMP9 substrates [41,42], but in quadruplicate experiments, the effects of MMP9 exposure on the relative staining intensities and electrophoretic profiles were more profound for VTN than DCN (Figure 1a) (Supplementary Table S1). This differential effect of MMP9 exposure was also evident by LC–MS/MS analysis. MMP9 cleavage sites in these two proteins were identified by performing in-gel digestion with trypsin, LC–MS/MS and a bioinformatic analysis pipeline, which searched for non-tryptic (assumed to be MMP9-derived) sites in the digested samples. Using this approach, we identified 33 putative cleavage sites in VTN (478 AA residues) compared with only 18 in DCN (359 AA residues) (Figure 1b). MMP9 cleavage sites in VTN were located primarily between AAs 300–400, corresponding to the heparin-binding domain, which has a proven involvement in fibronectin deposition [43]. In contrast, DCN cleavage sites were distributed throughout the AA sequence (Figure 1b). We next compared these experimentally determined cleavage sites with those predicted by PROSPER and DeepCleave. While PROSPER and DeepCleave achieve excellent performance against training available in MEROPS data (which represent diverse experimental approaches to establish enzymatic cleavage sites) their accuracy in predicting MMP9 cleavage sites in our controlled native VTN and DCN protein digests (as evaluated by area under the curve (AUC) score) was slightly better than random (PROSPER AUC: 0.55; DeepCleave AUC: 0.64) (Figure 1c).

In order to compare the predictive capabilities of PROSPER and DeepCleave against a complex proteome, we decellularized a post-confluent culture of HDFs and digested the remaining ECM with MMP9. Over 60 proteins were identified by LC–MS/MS in the baseline (non-MMP9 digested) matrix, many of which (COL6A1, COL6A2, COL6A3, EMILIN1, COL1A1, COL1A2) are found in the human dermis [44,45]. Putative MMP9 cleavage sites were identified in 40 out of 60 proteins, (including COL6A1, EMILIN1, FBLN2, COL1A2) resulting in 390 putative MMP9 cleavage sites (Figure 1d). Both PROSPER and DeepCleave performed poorly in accurately predicting cleavage sites in this complex mixture of native ECM proteins (PROSPER AUC: 0.56; DeepCleave AUC: 0.6) (Figure 1e). This may be due to: (i) a mismatch between the methods used to identify cleavage sites in the training and test data sets, or (ii) the lack of relevant training data in MEROPS (only 301 MMP cleavage sites derived from both intact protein and peptide substrates) [27]. A new protease cleavage site prediction model, which utilizes not only state-of-the-art machine learning approaches but also more expansive, up-to-date training datasets of native protein substrates, calibrated against experimental data, may achieve better accuracy.

### 2.2. Protease Cleavage Site Prediction Performance Can Be Improved Using a Deep Bidirectional Recurrent Neural Network Architecture

**Figure 2:**
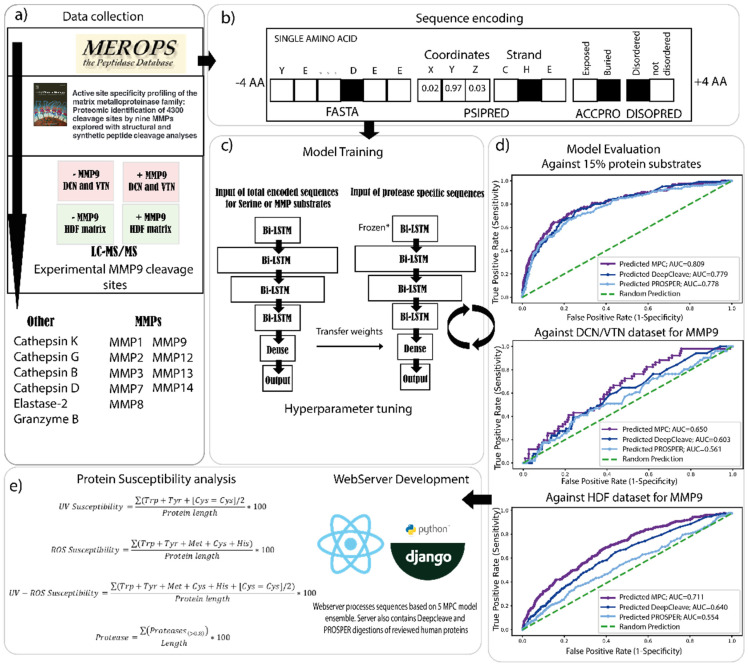
Manchester Proteome Cleave prediction model: architecture and testing. (**a**) We developed a novel protease prediction algorithm, the Manchester proteome cleave (MPC), based on state-of-the-art deep learning methodologies. The development of MPC involved five major steps: (**a**) data collection: integration of data from publicly available protease cleavage databases (MEROPs), the Eckhard 2016 dataset and our own in-house experiments. (**b**) Sequence encoding: MPC utilized primary AA sequence and structural features derived by in silico modeling with PSIPRED, DISOPRED and ACCPRO. One-hot encoding was used to represent the protein sequences in the format required for recurrent neural networks. (**c**) Model training: the MPC model consisted of 4 bidirectional long-short-term memory (Bi-LSTM) layers, one fully connected dense layer and an output layer. A general protease model was first pre-trained, and the resultant weights were transferred to train a protease-specific model. (**d**) Model evaluation: hyperparameters were tuned against 15% of excluded proteins and for MMP9, also against our experimental DCN/VTN and human dermal fibroblasts (HDF) cleavage site dataset utilizing a grid search strategy. (**e**) Webserver Development and Protein Susceptibility analysis: the best-performing architecture was used to train an additional four models, which were integrated into our MPSC web tool. Hence MPSC uses 5 models-averaged ensembles to infer the final cleavage scores for each of the proteases. MPSC web tool is also capable of performing UV and ROS assessments of proteins. The MPSC web server is publicly available on https://www.manchesterproteome.manchester.ac.uk/#/MPSC.

Recurrent neural network (RNN) architectures, initially developed for natural language processing, are particularly suited to analyze genomic and proteomic sequences [46]. This is particularly important for modeling proteolysis, as the AAs surrounding the cleavage sites play a significant role in determining the cleavage specificity [47]. RNNs have recently achieved notable success in the field of proteomics but have not yet been used to model proteolysis [35,48]. Here we adapted the methodologies employed by both PROSPER and DeepCleave to develop a novel, deep bidirectional recurrent neural network (deep BRNN) based proteolysis prediction algorithm calibrated against in-house experimental datasets. For data collection, the training, testing and validating datasets for serine proteases and MMPs were collected from the MEROPS database [27] and supplemented with additional MMP cleavage sites from a 2016 study [49] by Eckhard et al. Data were collected for two groups: MMPs (-1, -2, -3, -7, -8, -9, -12 and -13) and other proteases (Elastase 2, granzyme B, cathepsin -K, -G, -B, -D). These published datasets were also supplemented with the cleavage site information generated from our MMP9 LC/MS-MS experiments (Figure 2a).

In addition to the primary AA sequences, secondary structure, disordered regions and solvent accessibility may also play significant roles in determining the probability of protease cleavage [30]. Therefore, we used the methods previously employed by PROSPER (PSIPRED [50], DISOPRED2 [51] and ACCPRO 5.2 [52,53]) to predict these structural features for the whole human proteome prior to sequence encoding and model training. Each AA for every protein, in combination with these structural features, was encoded in a format suitable for RNNs. For sequence encoding, a sliding 8 AA window (4 AAs upstream and 4 AAs downstream of the predicted cleavage site) was utilized (Figure 2b).

To build a new cleavage site prediction model, we needed to address two challenges. First, there is limited data on MMP9 substrate specificity (e.g., MEROPs contain information on 301 cleavage sites from 53 protein substrates) for training deep learning models (Supplementary Figure S1). Second, the large imbalance between cleavage sites vs. non-cleavage sites (i.e., the number of non-cleavage sites vastly outweighs the number of cleavage sites) resulting in unbalanced datasets. The first challenge was addressed by (i) complimenting the MEROPS cleavage data with data available in the Eckhard 2016 study [49] and; (ii) using transfer learning approaches, where a general protease cleavage model was pretrained using all the available data (separately for MMPs and other proteases) and subsequently used as a starting point to train protease-specific models (using only the cleavage data for a specific protease) [54]. The second issue was addressed by weighting the model prior to model training to “pay more attention” to the minority (cleavage sites) class. For model training, different architectures, depths and hyperparameters of deep learning networks were evaluated in terms of area under the curve (AUC), the harmonic mean of the precision and recall score (F1) and The Matthews correlation coefficient (MCC) scores against cleavage sites. These were sourced from (i) protein substrate identities for each protease from the MEROPS and the Eckhard 2016 study, which were excluded from the training datasets (consisting of 15% of the total number, randomly selected); (ii) the DCN, VTN and HDF MMP9 LC–MS/MS cleavage site datasets as previously described. The best performing architecture was composed of four bi-LSTM layers, one dense layer and one fully connected layer (Figure 2c). Crucially, this setup was capable of predicting cleavage sites in ECM proteins more accurately compared to PROSPER and DeepCleave for all test sets evaluated by AUC, F1 and MCC scores. The AUC scores for MMP9 for: (i) the 15% protein test set were MPC: 0.809, DeepCleave: 0.779 and PROSPER: 0.778; (ii) the DCN and VTN test set were MPC: 0.650, DeepCleave: 0.603 and PROSPER: 0.561 and; (iii) the HDF data were MPC: 0.71, DeepCleave: 0.640 and PROSPER: 0.554 (Figure 2d), respectively. For each of the proteases, there was some agreement between MPC, DeepCleave, and PROSPER predicted cleavage sites; however, each algorithm also identified cleavage sites unique to the model (Supplementary Table S2). Tables of AUC values and 95% confidence intervals are reported in Supplementary Figure S2.

### 2.3. Development of a Web Tool to Predict Protein Susceptibilities: Step #1 Amino Acid Composition

**Figure 3:**
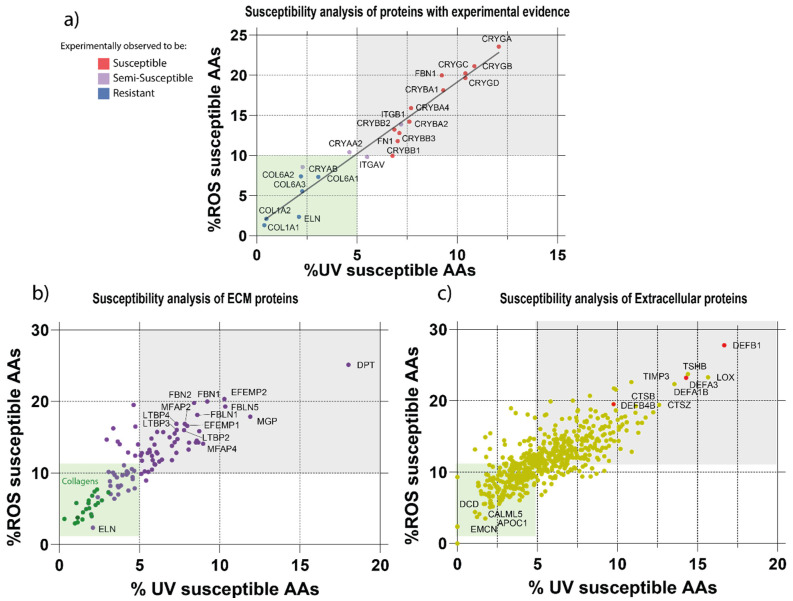
Predicted susceptibilities of skin proteins to photo- and oxidative-damage based on amino acid (AA) composition. (**a**) Predicted ultraviolet radiation (UVR) and/or ROS susceptibilities of proteins previously reported as susceptible or resistant by experimental observation. Experimentally verified UVR and ROS susceptible proteins (red) are enriched in UVR- and ROS-susceptible moieties compared with resistant proteins (blue). The predicted UVR- and ROS-susceptibilities exhibited a positive linear correlation (R^2^ = 0.94). Applying thresholds to distinguish between experimentally susceptible and resistant proteins suggests that a composition of >5% UVR AA residues and >10% oxidation-sensitive AA residues may be indicative of UV/ROS susceptibility (susceptible = gray box, resistant = green box). (**b**) Predicted UVR- and ROS-susceptibilities of skin’s ECM proteins. Elastic fiber-associated proteins, except for elastin itself, and collagens (green) clearly stratify into susceptible and resistant risk categories, respectively. (**c**) Predicted UVR and ROS susceptibility of non-ECM extracellular proteins. Defensins (red) are highlighted as an example of a susceptible protein family.

Having developed a new protease cleavage site prediction model, we next sought to develop a web tool to predict protein susceptibly to proteolysis by multiple mechanisms. The first step was to expand our previous analyses of amino acid composition (and hence UVR/ROS susceptibility) [12] to the whole skin proteome. The AA residues Trp, Tyr and double-bonded Cys (Cys = Cys) are sensitive to both biologically relevant wavelengths of UVR and oxidation [14], whereas Met, His, and Cys are sensitive to oxidation alone [55]. We have previously established, in a limited survey of skin components, that proteins rich in these AA residues are greater at risk of degradation by UVR and/or ROS [7]. Building on these insights, we have now reviewed published data from experimental studies, which subjected ECM proteins and assemblies to physiologically relevant UVR doses and wavelengths, and categorized selected proteins as susceptible, semi-susceptible or resistant (Supplementary Table S1). As AA composition can predict the relative susceptibilities of these proteins to UVR/ROS-mediated proteolysis (Figure 3a), we have integrated this approach into the MPSC web tool.

Applying the MPSC to skin ECM proteins (as defined by the Manchester skin proteome) predicts that many elastic fiber-associated proteins (FBN1, FBN2, FBLN1, FBLN5, LTBP2, LTBP3, LTBP4, EFEMP2, MFAP2 and MFAP4) [56,57] will be highly susceptible to degradation by both UVR and oxidation (Figure 3b). These predictions agree well with in vivo observations of dermal remodeling in photoaging where the loss of FBN1 and FBLN5 from the papillary dermis, and the presence of disorganized material termed solar elastosis, characterizes mildly and severely photoaged skin, respectively [58,59,60]. In contrast, based on AA composition, skin collagens are likely to be relatively resistant to both UVR and to ROS-mediated oxidation (Figure 3a,b), and previously, we have shown that the electrophoretic mobility of collagen I alpha chains, the most abundant skin proteins, are unaffected by physiologically relevant doses of UVR [12]. However, in vivo, observational studies show that collagen I abundance decreases in aged (and particularly in photoaged) human skin [61,62], suggesting that other mechanisms may drive collagen remodeling.

In addition to predicting the relative UVR/ROS susceptibilities of major structural components, such as elastin and the collagens, our analysis also identifies potential novel markers involved in the regulation of TGFβ and collagen fibril formation (DPT) and ECM development (MGP), which, to our knowledge, have not previously been implicated in photoaging (Figure 3b). Extracellularly, MPSC predicts that the defensin family (DEFB1, DEFA3, DEFA1B and DEFB4B) of proteins may be UVR/ROS susceptible (Figure 3c). This is consistent with the ability of defensins to act as antioxidant proteins [63]. Crucially, MPSC not only predicts that known photoaging candidate biomarkers (LOX, TIMP3) will be susceptible to UVR/ROS degradation [44,64,65] but also highlights the potential susceptibility (TSHB, CTSB, CTSZ) and resistance (DCD, EMCN, CALM5 and APOC1) of new skin aging biomarker candidates (Figure 3c). In complex tissues, degradative mechanisms do not act in isolation [36,37], and we, and others, have shown that UVR exposure can enhance protease-mediated-proteolysis [7,66].

### 2.4. Development of a Webtool to Predict Protein Susceptibilities: Step #2 Integration of Protease Cleavage Site Prediction Models

MPSC predicts relative protease susceptibility as the number of protease cleavage sites/protein length. Cleavage site predictions are derived from an ensemble model averaging five MPC model outputs (Figure 2e). This approach is based on the hypothesis that cleavage site abundance predicts experimental protease susceptibility. In order to test this hypothesis, we compared cleavage site predictions with experimental detection by LC–MS/MS. Characterization of the HDF-derived matrix revealed two groups of proteins, those with and those without experimentally detected MMP9 cleavage sites. Proteins apparently devoid of MMP9 cleavage sites included COL3A1, COL12A1, LTBP2, VCAN and sixteen others (Figure 1d). We have used the identities of these cleaved and non-cleaved proteins to determine whether predicted MMP9 protease susceptibilities differ statistically between these two groups. The MPSC score for MMP9 was set to 0.8, which corresponds to highly confident cleavage sites. Using this approach, proteins with not experimentally detected MMP9 cleavage in the HDF-derived proteome also had significantly lower predicted MMP9 susceptibilities than the proteins that had at least one MMP9 cleavage site (*p* = 0.005, Student’s *t*-test). This analysis suggests that novel substrates for proteolytic degradation may be identified using in silico proteolytic modeling and supports the hypothesis that predicted cleavage site abundance is an indicator of relative susceptibility.

We next applied MPSC web tool calculations to every ECM and extracellular protein in the Manchester skin proteome [45] in order to predict their susceptibility to UVR/ROS and to UV-upregulated skin proteases (interstitial collagenase: MMP1, 92 kDa gelatinase: MMP9, stromelysin-1: MMP3, cathepsin K, and granzyme B) [40,67].

Using the MPSC web tool, we initially analyzed skin ECM proteins for their predicted protease and UVR/ROS susceptibilities using MPC models. This analysis suggests that tropoelastin (the elastin precursor) will be particularly susceptible to MMP-mediated cleavage, which agrees well with experimental observations [68]. In addition, multiple proteins involved in elastic-fiber formation and organization (FBLN1, FBLN2, FBLN5 EFEMP1, EFEMP2, MFAP4 and FBN2) were also predicted to be susceptible to not only MMP proteolysis but also to UVR/ROS. These predictions suggest that both proteases-mediated and UVR/oxidative damage may play a role in the deterioration of the elastic fiber architecture, which is characteristic of photodamage in human skin [69]. We have previously shown an interplay between these mechanisms whereby UVR exposure enhances the degradation of fibrillin microfibrils [66].

In addition to elastic fibers, collagens also undergo profound remodeling in photo-exposed skin. Protease-mediated activity has long been suspected as a driver of age-related degradation of skin collagens [61,62], but given the intermittent and/or chronic low-level action of these mechanisms, the causative link has yet to be established. Whilst the protease susceptibility of Collagen I and III has been confirmed experimentally [70], our computational techniques also suggest that many skin collagens (i.e., II, III, IV, V, VI, XII, and XV) will be degraded by proteases (MMP-1, -3, -9, granzyme-B, and cathepsin K), but not by UVR/ROS. For the ubiquitous microfibrillar collagen VI assemblies, protein abundance and architecture is resistant to in vivo photodamage [16], suggesting that this ECM assembly is resistant to multiple degradative agents. However, the alpha 5 chain of COL6 is degraded by cathepsin K [71,72], and we have shown subtle UV-induced changes in the structure-associated features by mass spectrometry [15]. Therefore, we conjecture that differential mechanisms may drive the degradation of elastic fiber components and skin collagens, necessitating different preventative and/or therapeutic approaches.

**Figure 4:**
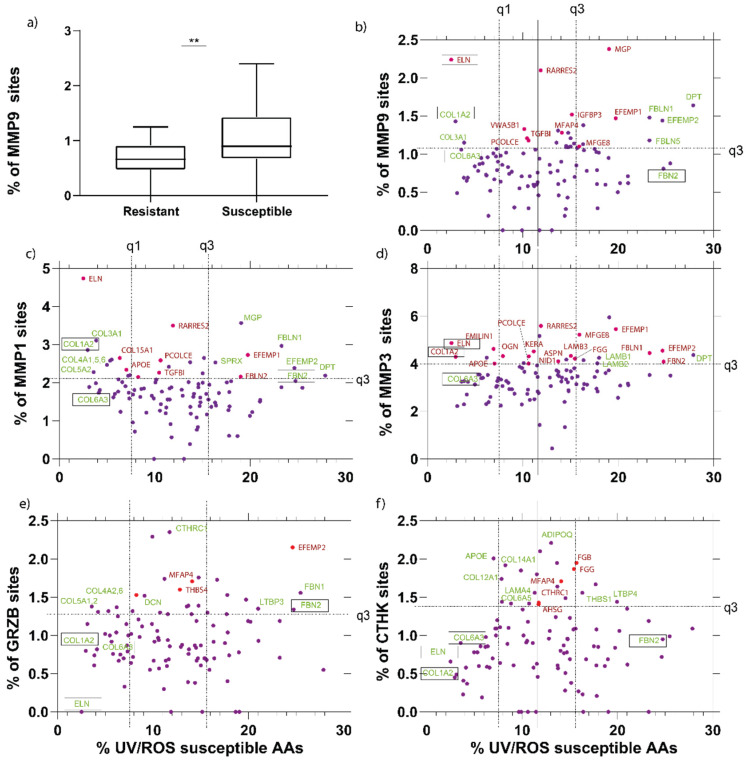
UVR/ROS vs. protease susceptibilities of MMP1, 3 and 7, granzyme B and cathepsin K of skin proteome-derived extracellular matrix (ECM) components. (**a**) Using the protein identities in the MMP9 degradation dataset, which had no MMP9 cleavage sites or had at least one cleavage site, allowed the evaluation of the MPC model’s performance to predict novel substrates. Proteins that had no experimental cleavage sites yielded significantly lower predicted MMP9 susceptibility scores. Having defined that susceptibilities could be analyzed using the MPC model, we used these models and analyzed the susceptibilities for MMP1 (**c**), 3 (**d**), and 9 (**b**), granzyme B (**e**), and cathepsin K (**f**). Proteins that were predicted as highly susceptible by PROSPER or DeepCleave only are labeled in red text. Proteins that are predicted as highly susceptible by MPC only are labeled in green text. Throughout, we have highlighted (boxed text) important skin proteins COL1A2, FBN2, ELN and COL6A3, which merit discussion.

In addition to predicting the relative susceptibility of major structural ECM assemblies, our analysis also suggests that protease-mediated proteolysis of basement membrane proteins, including laminins (LAMB-1, -2, -3, -4, LAMA-3, -4) and nidogen (NID1), may be a contributing factor to the remodeling of the dermal-epidermal junction in photo-exposed skin [73]. Other proteins predicted to be highly susceptible to MMP degradation include several proteoglycans (OGE, ASPN, KERA, and DPT), retinoic acid receptor responder protein 2 (RARRES2), matrix Gla protein (MGP), procollagen C proteinase enhancer (PCOLCE), TGFβ1, apolipoprotein E (APOE) and Von Willebrand factor (VWA5B1). Some proteins appear to be susceptible to only a single enzyme, such as collagen triple helix repeat-containing protein 1 (CTHRC1: granzyme B) and adiponectin receptor protein 1 (ADPOQ: cathepsin K) (Figure 4b–f).

**Figure 5:**
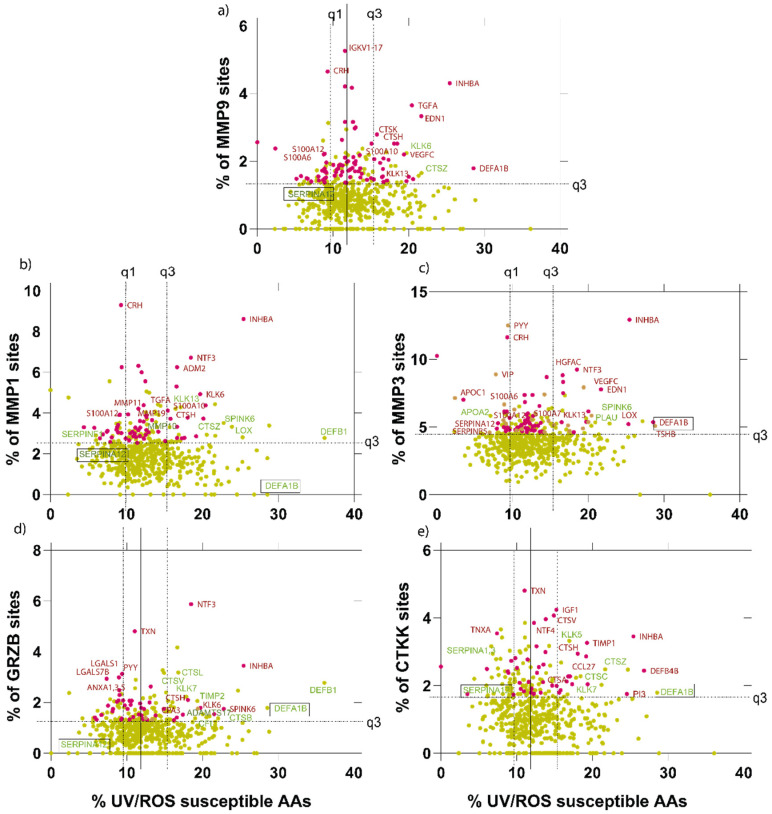
UVR/ROS vs. protease susceptibilities of MMP1, 3 and 7, granzyme B and cathepsin K of skin proteome-derived extracellular components. Similar to the ECM components, we have also investigated proteins in the extracellular space important in maintaining the ECM. We analyzed extracellular components for MMP1 (**b**), 3 (**c**), 9 (**a**), granzyme B (**d**) and cathepsin K (**e**). Proteins that were predicted as highly susceptible by PROSPER or DeepCleave only are labeled in red text. Proteins that are predicted as highly susceptible by MPC only are labeled in green text. Throughout, we have highlighted (boxed text) important skin proteins, which merit discussion.

Photoaging may also affect non-structural extracellular proteins, such as growth factors, antioxidants and enzymes, which play key roles in the maintenance and regeneration of normal healthy skin. Our analysis suggested that MMPs, cathepsin-K and granzyme-B are capable of digesting (or self-digesting) each other in addition to other catalytic proteins and inhibitors, such as cathepsins themselves (A, C, K, H, B, Z, V), Kallikreins (KLK5, KLK6, KLK7, KLK13), MMPs (MMP10, MMP11, MMP19, CPA3), PLAU, ADAMTS17, CFI, and protease inhibitors TIMP1 and 2, SPINK6, CST6, PI3, SERPINF1, SERPINA1, 3, 12, SERPINB5. It has been previously shown that proteases exhibit complex synergetic relationships, cleaving one another to amplify protease cascades, such as those mediated by the so-called “cysteine switch”. [74]. This consistent prediction of such catalytic proteins being protease-susceptible markers and the extensive literature of protease activation mechanisms strengthens our confidence in the predictions of the MPC. Defensin family members (DEFA1B, DEFB1, DEFB4B) - which play antimicrobial roles in skin - are predicted by the MSPC to be highly susceptible to both UVR/ROS and proteases, thereby suggesting their role not only as antimicrobial peptides but also potential sacrificial sunscreen-like proteins and antioxidants [63,75]. Other examples of proteins predicted to be susceptible to both proteolysis and UV/ROS include IGF1, TGFα, INHBA, NTF3, VEGFC, HGFAC, TSHB, and SLURP1. Markers predicted to be susceptible to proteolysis but resistant to UVR/ROS include galectins (LGALS1, LGALS7B, LGALSL) and annexins-1, 3, 5 (ANXA1, ANXA3, ANXA5), potentially indicating their need to be resistant to environmental factors (Figure 5a–e).

## 3. Materials and Methods

### 3.1. Dataset Collection

FASTA sequences and disulfide bonds for each protein in the Manchester skin proteome (MSP) were retrieved from the Uniprot database in Jan 2020 [76]. For MPSC development, experimentally validated protein substrate annotations were collected from Eckhard 2016 and MEROPS (v12.2) in Jan 2020 [27,49]. Only human protein cleavage sites were retrieved. The entire MSP [45] and proteins present in the Eckhard 2016 were digested using locally installed PROSPER, and full proteome digestion by DeepCleave was provided by our Monash collaborators.

### 3.2. Deep RNN Protease Model

#### 3.2.1. Evaluation Metrics

As the dataset is highly imbalanced, containing thousands of non-cleavage sites vs. only hundreds of cleavage sites, and thus in order to evaluate the performance of PROSPER, DeepCleave and MPC, we used F1 score representing the harmonic mean of precision and recall; the Matthews’ correlation coefficient (MCC), precision, recall and area under the curve (AUC) scores. Performance differences of classifiers are visualized with receiver operating characteristic (ROC) curves (sensitivity-(true positive rate) against the 1-specificity (false-positive rate) and evaluated with area under the curve (AUC) score. MCC and F1 scores are defined as follows:

(1) $$\mathrm{MCC}=\frac{\mathrm{TP}*\mathrm{TN}-\mathrm{FP}*\mathrm{FN}}{\sqrt{\left(\mathrm{TP}+\mathrm{FP}\right)\left(\mathrm{TP}+\mathrm{FN}\right)\left(\mathrm{TN}+\mathrm{FP}\right)\left(\mathrm{TN}+\mathrm{FN}\right)}}$$

(2) $$F1\;\mathrm{score}=\frac{2\mathrm{TP}}{2\mathrm{TP}+\mathrm{FP}+\mathrm{FN}}$$

where TP represents the number of true positives, TN represents the number of true negatives, FN represents the number of false-negatives, and FP represents the number of false-positives, respectively.

#### 3.2.2. Feature Extraction

PROSPER revealed that not only the AA sequence context surrounding cleavage sites, but also protein secondary structure, disordered regions and solvent accessibility play important roles in proteolytic cleavage site prediction [30]. The whole human proteome was annotated with PSIPRED [50], DISOPRED2 [51] and ACCPRO 5.2 [52,53] to retrieve these different types of sequence-derived structural features.

#### 3.2.3. One-Hot Encoding

Each AA was encoded using the one-hot encoding, resulting in a 20-dimensional vector where each dimension represents one of the 20 common AAs. To gain insights from the surrounding AAs, a sliding window of −4 and +4 AAs additional to the cleavage site was used for each sequence. At the N- and C- termini, where there are no following AAs, each position of the 20-dimensional vector contained only zeros. Furthermore, we complemented each of these vectors with three-dimensional coordinates retrieved from SCRATCH (x, y, z), one hot-encoded representation of whether amino acid was part of the coil, strand or helix, two-dimensional one-hot vector encoding whether it was exposed or buried, as well as a two-dimensional one-hot vector of whether AA was disordered or not.

#### 3.2.4. Train, Test and Validate Data Split

In contrast with previously published models, which split the data in train, test and validate datasets on the cleavage site basis regardless of the protein source, we have ensured that the testing, training and validating data all come from independent proteins. We used TensorFlow random seed to ensure the consistency of evaluations and ensured that the same protein identities were selected each time when evaluating the model performance; 70% of the proteins were used for the training, 15% for testing and 15% for validating.

#### 3.2.5. Architecture of the Deep RNN

We used the Python 3.8 TensorFlow Keras package to implement our MPC model. There are many more non-cleavage sites than the cleavage sites resulting in a very imbalanced dataset. To overcome this, we assigned larger weights to cleavage sites than non-cleavage sites, enforcing the classifier to “pay more attention” to the underrepresented class. We also utilized transfer learning to overcome the issue with limited amounts of data available for certain proteases [54]. We ensured that the general model and protease-specific model contained the same identities for testing, training, and validating. MPC protease models consist of four bidirectional LSTM layers, a fully connected Dense layer and an output layer. We set epochs to a very large number (>10 000) and monitored the early stopping by measuring the maximum F1 score with a patience of 20 epochs. A generic model utilized all the MMPs or other proteases. We then used transfer learning to convert the generic model into the protease-specific model by loading the initial weights, freezing the initial layer and continuing the training process with the data that only had protease-specific entries. Our experimental DCN/VTN and HDF MMP9 datasets were used to tune the hyperparameters (the number of AAs surrounding cleavage site, learning rate, batch size, etc.) to achieve the optimal performance in the skin-relevant model system. Subsequently, based on the optimized hyperparameters, we trained 5 models for each of the proteases and took the average output score as the final prediction.

### 3.3. UVR/ROS and Protease MPSC Susceptibility Model Calculations

Previous analysis has revealed a clear agreement between experimental UV and ROS susceptibility and chromophore content of the FASTA sequence. To implement the UV, ROS and combined UVR/ROS model, simple mathematical equations were derived, which quantify the number of UV and ROS chromophore AAs per length of the sequence. For proteolysis susceptibility calculations, highly confident (score > 0.8), MPC cleavage sites were selected, and the number of cleavage sites per protein length calculated. The calculations employed in MPSC are:

(3) $$\mathrm{UV}\;\mathrm{susceptibility}=\frac{\sum (\mathrm{Trp}+\mathrm{Tyr}+\left[\mathrm{Cys}=\mathrm{Cys}\right]/2)}{\mathrm{Protein}\;\mathrm{length}}*100$$

(4) $$\mathrm{ROS}\;\mathrm{Susceptibility}=\frac{\sum (\mathrm{Trp}+\mathrm{Tyr}+\mathrm{Met}+\mathrm{Cys}+\mathrm{His})}{\mathrm{Protein}\;\mathrm{length}}*100$$

(5) $$\mathrm{UV}-\mathrm{ROS}\;\mathrm{Susceptibility}=\frac{\sum (\mathrm{Trp}+\mathrm{Tyr}+\mathrm{Met}+\mathrm{Cys}+\mathrm{His}+\left[\mathrm{Cys}=\mathrm{Cys}\right]/2)}{\mathrm{Protein}\;\mathrm{length}}*100$$

(6) $$\mathrm{Protease}=\frac{\sum \left({\mathrm{Proteases}}_{\left(>0.8\right)}\right)}{\mathrm{Protein}\;\mathrm{length}}*100$$

where Trp represents tryptophan, Tyr represents tyrosine, Met represents methionine, Cys represents cysteine, His represents histidine, and Cys = Cys represents disulfide bound cystine.

### 3.4. LC–MS/MS Methods for Generating Experimental Testing Dataset

#### 3.4.1. Cell Culture

Human dermal fibroblasts (HDFs) were cultured from a scalp biopsy obtained from a hair transplant procedure. Ethical approval was obtained from the National Research Ethics Committee (ref 19/NW/0082), and the tissue donor provided written, informed consent prior to the procedure. Skin biopsies were cultured dermis-side down in Dulbecco’s modified Eagle’s medium (DMEM) (Thermo Fisher, Waltham, MA, USA) containing 20% (*v*/*v*) fetal bovine serum (FBS), GlutaMAX (2 mM), penicillin (100 U/mL), streptomycin (0.1 mg/mL) and amphotericin-B (2.5 µg/mL) until HDFs were observed migrating out of the explant. The culture medium was changed every 2–3 days until cells reached 80% confluence (approximately 3 weeks), at which point explanted tissue was aseptically removed from the culture and HDFs were subcultured using trypsin (0.05% *w*/*v*)-ethylenediaminetetraacetic acid (EDTA; 0.02% *w*/*v*). Subsequent culture of HDFs used supplemented DMEM containing 10% (*v*/*v*) FBS. For extracellular matrix (ECM) deposition, HDFs at passage 4 were seeded at 1.2 × 10^5^ cells/mL, in 0.5 mL medium containing 50 µg/mL ascorbic acid (final concentration), in a 24-well plate, and cultured until confluence (approximately 2 days). At this point, a 0.25 mL medium was removed and replaced with a fresh medium. The medium was replaced every 2–3 days.

#### 3.4.2. HDF-Deposited ECM In Vitro

The procedure for obtaining HDF-deposited ECM was carried-out as previously described [77]. Briefly, the medium was removed from cultures on day 9 post-confluency, and the cell layer washed twice with phosphate-buffered saline (PBS). To remove cells, 0.5 mL of extraction buffer (20 mM ammonium hydroxide solution, 0.5% *v*/*v* Triton-X 100, in PBS without Ca^2+^/Mg^2+^) was added to HDFs for 5 min, and cell depletion was determined by repeatedly checking cultures under a light microscope. Extraction buffer was diluted by the addition of PBS, and HDF-depleted ECM was incubated at 4 °C overnight. Following the removal of diluted extraction buffer, the remaining cell debris was removed by three successive washes in PBS. The presence of deposited, HDF-depleted ECM was determined by light microscopy.

#### 3.4.3. MMP9 Degradation of ECM and DCN/VTN In Vitro

Human recombinant MMP9 (hrMMP9; R&D Systems) was activated by adding 1 mM (final concentration) ρ-aminophenylmercuric acetate (APMA) and incubating for 24 h at 37 °C. HrMMP9 activity was confirmed by measuring cleavage of the fluorogenic peptide, Mca-PLGL-Dpa-AR-NH_2_ (R&D Systems), according to the manufacturer’s instructions. HDF-depleted ECM was incubated with 10 µg/mL of active hrMMP9, whereas DCN (ab167743, Abcam, Cambridge, UK) and VTN (ab94369, Abcam) was incubated at a 1:2 protein to active hrMMP9 ratio, diluted in MMP buffer (50 mM Tris, 150 mM NaCl, 10 mM CaCl_2_ and 0.05% (*w*/*v*) Brij-35, pH 7.5) at 37 °C with agitation, for 24 h. For HDF ECM assay, the buffer was removed and dialyzed for 4 h against ddH_2_O (Thermo Fisher), and samples freeze-dried overnight. DCN and VTN samples were run on 4–12% NuPAGE Novex Bis-Tris Gels (1 µg of protein per well) using standard SDS–PAGE procedures. Nondigested control bands and digested bands were excised using a scalpel blade. ECM or DCN and VTN incubated with MMP buffer alone served as negative protease controls, respectively.

#### 3.4.4. HDF-Deposited ECM Sample Preparation

Each sample’s aliquot was added to SMART Digest™ trypsin (Thermo Fisher) beads and shaken at 1400 rpm overnight at 37 °C. The next day, reduction and alkylation were performed. Next, SMART digest beads were removed using TELOS MicroPlate™ filter tips (Kinesis; Cheshire, UK) into 1.5 mL Eppendorf^®^ LoBind tubes (Eppendorf; Stevenage, UK) to minimize electrostatic loss of peptides and acidified with 5 µL of 10% formic acid (FA). Biphasic extraction was performed to partition organic molecules and surfactants. To do this, 200 μL of ethyl acetate (EA; Sigma, St. Louis, MO, USA) was added to each sample in Eppendorf^®^ LoBind tubes and vortexed for 1 min. Samples were then centrifuged to ensure phases were separated and the upper layer removed using gel-loading pipette tips. These steps were repeated twice with a further 200 μL of EA, and then the resultant aqueous bottom phase was vacuum dried for 2 h at room temperature. Once dry, 200 μL of injection solution (5% (*v*/*v*) acetonitrile, 0.1% formic acid) was added to the tube, and peptide desalting was performed. For all desalting of peptide preparations, OLIGO™ R3 reversed phase resin (Thermo Fisher) beads were used. Beads were wetted to be activated using 50 μL OLIGO™ R3 beads plus 50 μL of wet solution (50% acetonitrile in ultrapure water; 1:1). Twenty (20) μL of this mix were placed in detached TELOS filter tips, and the liquid was pipetted out using a standard p1000 Gilson pipette (Gilson; Middleton, WI, USA) and the process repeated twice more. Twice beads were washed by adding 50 μL of wash solution (0.1% FA in ultrapure water) and liquid pipetted out. Once the beads were activated, 100 μL of the sample was gently added to TELOS filter tips containing activated OLIGO R3 beads, allowing the peptides from the sample to stick to the beads. The liquid was pipetted out, and the “flow-through” was retained. The process was repeated with the remaining sample. Twice beads with bound peptides were gently resuspended in 50 μL of wash solution, and the flow-through was discarded. Twice, beads were gently suspended in 50 μL of eluting solution (50% (*v*/*v*) acetonitrile, 0.1% formic acid) to release the peptides from the beads and liquid was pipetted out and retained. The collected eluted sample contained the peptides for analysis. Eluted samples were transferred to mass spectrometry vials and vacuum dried using Speed Vac (Heto-Holten; Frederiksborg, Denmark), ensuring peptide stability until mass spectrometry was available.

#### 3.4.5. DCN and VTN Gel Sample Preparation

Excised bands were first placed into a perforated well plate and then shrunk by washing in acetonitrile for five minutes. To remove the acetonitrile from the samples, these were centrifuged for one minute at 1500 rpm. Sample gel pieces were then dried in a vacuum centrifuge for 15 min. Next, samples were covered completely by 10 mM dithiothreitol (DTT) in 25 mM ammonium bicarbonate and incubated at 56 °C for one hour to reduce the proteins. Samples were cooled to room temperature, then centrifuged to remove the DTT. Iodoacetamide (55 mM) in 25 mM ammonium bicarbonate was added, and the samples were incubated for 45 min in the dark at room temperature. The iodoacetamide solution was removed by centrifugation, and the samples were washed with 25 mM ammonium bicarbonate for 10 min. Samples were then washed with acetonitrile, followed by another wash with ammonium bicarbonate, then a final wash with acetonitrile, centrifuging in-between steps to remove the previous wash. The samples were then centrifuged again, then dried out in a vacuum centrifuge. Next, 5 μL of a 12.5 ng/μL solution of trypsin along with 45 μL of a 25 mM ammonium bicarbonate solution was added to the dried samples. Samples were kept at 4 °C for 45 min for samples to absorb the buffer. After this time, samples were covered completely in 25 mM ammonium bicarbonate. Samples were incubated at 37 °C overnight. The following day, peptides were extracted by incubating with 20 mM ammonium bicarbonate for 20 min, then centrifuging into a fresh storage plate. Peptides were extracted a further two times (20 min each) using 5% formic acid in 50% acetonitrile, using the same storage plate to pool all the supernatant into the same tube per sample. Samples were transferred to mass spectrometry vials and vacuum-dried using Speed Vac (Heto-Holten; Frederiksborg, Denmark).

#### 3.4.6. Peptide Preparation for Mass Spectrometry

For LC–MS\MS analysis, 20 µL of Injection Solution (5% acetonitrile (ACN) + 0.1% formic acid (FA) in ultrapure water) was added to each dried peptide samples. Peptide concentration in solution was measured using a Direct Detect infrared spectrometer (Millipore). According to the concentration detected for each sample, they were diluted to have 12 µL of the solution with a concentration of 800 ng/µL, and samples were submitted to the Biological Mass Spectrometry Core Facility (University of Manchester).

#### 3.4.7. Liquid Chromatography–Tandem Mass Spectrometry

Mass spectrometry was performed according to the Facility’s protocols [78,79]. Digested samples were analyzed by LC–MS/MS using an UltiMate^®^ 3000 rapid separation LC (RSLC, Dionex Corporation, Sunnyvale, CA, USA) coupled to a Q Exactive HF (Thermo Fisher Scientific) mass spectrometer. Peptide mixtures were separated using a multistep gradient from 95% A (0.1% FA in water) and 5% B (0.1% FA in acetonitrile) to 7% B at 1 min, 18% B at 58 min, 27% B in 72 min and 60% B at 74 min at 300 nL min-1, using a 75 mm × 250 μm i.d. 1.7 µM CSH C18, analytical column (Waters). Peptides were selected for fragmentation automatically by data-dependent analysis.

### 3.5. Webserver Development and Data Visualization

The MPSC web tool was developed using the ReactJS framework for the front end and Python Django as the backend for the API development. Data powering the MPSC web tool was stored on the Apache server on the MySQL database. Data for this publication were visualized using GraphPad Prism 8, Adobe Illustrator and Python Matplotlib libraries.

## 4. Conclusions

Degradomics is an ever-expanding field with the potential to impact translational research by deepening our understanding of tissue regeneration processes as well as contributing to drug discovery efforts [9,80]. In this work, we show that: (i) state-of-the-art deep BRNN techniques are capable of predicting proteolytic cleavage sites in native proteins with a better degree of accuracy than previously achieved and that (ii) combining predictions of protease and oxidative damage may identify novel pathological mechanisms and therapeutic targets. With this work, we have stratified proteins within the entire human skin proteome to reveal their predicted susceptibilities to UV, ROS and protease-mediated proteolysis providing significant novel insights in skin research. Particularly, we have highlighted the fact that fibrillar collagens are predicted to be preferentially degraded by proteases alone, whereas elastic fibers are likely to be susceptible to UVR/ROS and proteases in combination. We also demonstrate that proteins involved in catalytic processes have a high percentage of predicted confident cleavage sites per protein length. Moreover, we have made this analysis applicable to any AA sequence of interest. Beyond the field of dermatology, the concordance between our computational analyses and previously reported observations of both in vitro and in vivo protein degradation suggests that this approach has the potential to identify novel protein candidate biomarkers for tissues subjected to inflammation or aging-related disease.

Whilst the MPC model performed better than existing models, its success in predicting experimental cleavage sites in native proteins still requires further improvement. Furthermore, while we have attempted initial experimental MMP9 cleavage site detection in a complex HDF proteome using LC–MS/MS approaches, it is clear that many cleavage sites may have been missed, particularly for low abundance proteins, where the peptide-coverage of the protein is also low; therefore, experimental approaches and sample preparation methods require even further improvement. The difficulty in predicting cleavage sites in native proteins may be partly due to the heterogeneous nature of the data currently available in public databases, such as MEROPS upon which the algorithms were built. For example, elastin degradation by proteases is very well-defined, but well-known MMP substrates (such as FBN1) are not necessarily reflected in the available databases [27,68,81]. Furthermore, data referenced in these databases is drawn from multiple experimental methods applied to disparate biological samples, including peptide libraries, extracted cartilage proteins, post mortem brain tissue, and many others, which may not necessarily translate to different in vitro systems [27]. Critically, we have shown using an LC–MS/MS peptide location fingerprinting approach that ECM proteins can exhibit tissue-dependent structures [82]. Furthermore, higher-order structures play a crucial role in determining proteolysis; however, currently, the accurate prediction of protein secondary and tertiary features remains challenging [83]. In this context, improved algorithms trained on expanded experimental datasets with additional informative features would benefit proteolytic cleavage site prediction. Although in its current form, the web tool is limited to predicting cleavage by 14 proteases (8 MMPS, 4 cathepsins, elastase-2 and granzyme B), given the availability of suitable training data, the approach used in this study could be applied to additional proteases. The improved prediction could also be achieved by taking into consideration the impact of local protein structures on protease-specific cleavage outcomes.

Overall, the MPSC web tool builds upon the success of the Prosper and DeepCleave algorithms, which have contributed significantly to the degradomics fields and can further assist in novel biomarker discovery, reveal the primary AA sequence susceptibilities to UV/ROS and proteases and even assist in novel bioactive matrikine discovery [84].

## Data Availability

Mass spectrometry data will be uploaded to the PRIDE repository.

## Supplementary Materials

The following are available online at https://www.mdpi.com/1422-0067/22/6/3071/s1, Figure S1: Western Blots; Figure S2: MPC, DeepCleave, Prosper model evaluations, Table S1: Literature evidence of protein degradation, Table S2: Experimental cleavage sites used for model training.
